# Construction, Investigation and Application of TEV Protease Variants with Improved Oxidative Stability

**DOI:** 10.4014/jmb.2106.06075

**Published:** 2021-09-10

**Authors:** Enkhtuya Bayar, Yuanyuan Ren, Yinghua Chen, Yafang Hu, Shuncheng Zhang, Xuelian Yu, Jun Fan

**Affiliations:** School of Life Science, Anhui Agricultural University, Hefei, Anhui 230036, P.R. China

**Keywords:** TEVp, mutation, cysteine residues, oxidative stability, tag removal, *Escherichia coli*

## Abstract

Tobacco etch virus protease (TEVp) is a useful tool for removing fusion tags, but wild-type TEVp is less stable under oxidized redox state. In this work, we introduced and combined C19S, C110S and C130S into TEVp variants containing T17S, L56V, N68D, I77V and S135G to improve protein solubility, and S219V to inhibit self-proteolysis. The solubility and cleavage activity of the constructed variants in *Escherichia coli* strains including BL21(DE3), BL21(DE3)pLys, Rossetta(DE3) and Origami(DE3) under the same induction conditions were analyzed and compared. The desirable soluble amounts, activity, and oxidative stability were identified to be reluctantly favored in the TEVp. Unlike C19S, C110S and C130S hardly impacted on decreasing protein solubility in the BL21(DE3), but they contributed to improved tolerance to the oxidative redox state in vivo and in vitro. After two fusion proteins were cleaved by purified TEVp protein containing double mutations under the oxidized redox state, the refolded disulfide-rich bovine enterokinase catalytic domain or maize peroxidase with enhanced yields were released from the regenerated amorphous cellulose via affinity absorption of the cellulose-binding module as the affinity tag.

## Introduction

As an enzymatic reagent, TEVp is often used for specific proteolysis of fusion protein at the recognition site ENLYFQG/S (tevS) placed between fusion tag and target protein to allow an extra glycine or serine left at N-terminus of target protein. Fusion tags function as a solubility enhancer, and affinity partner for increasing protein solubility in *Escherichia coli*, and rapid purification. In certain cases, the fused carrier affects enzyme activity and protein crystallization, and may produce unnecessary immunological reactions [1]. The wild-type TEVp displays several advantages, like high sequence specificity, functional production in *E. coli*, and less sensitivity to a relatively broad range of pH (6.0-9.0) or temperature (4.0-37.0°C) [2]. For improving protein solubility, several amino acid residues are mutated, such as L56V and S135G [3], T17S, N68D and I77V [4]. The introduced S219V, S219N, or S219P significantly inhibits the TEVp self-proteolysis near its C-terminus for auto-inactivation. Also, as a thiol protease, TEVp is sensitive to the oxidative environment because the thiol group in Cys151 responsible for catalysis is prone to be oxidized [5]. Besides Cys151, the TEVp contains C19, C110 and C130. When it is overexpressed in the endoplasmic reticulum of HEK-239T cells, the autolysis-deficient TEVp variant is inactivated, but its activity is rescued by N23Q, C130S and T173G mutations, but not recovered by the sole C130S. The activity of the constructed TEVp variant was blocked by additional C19S or C110S [6]. Crystal structures have revealed that C130 is involved in either forming an intermolecular disulfide bridge or binding with the reducing reagent β-mercaptoethanol [7, 8]. Mutation of the cysteine residue into one of serine or alanine is a common practice to improve protein stability in the oxidative environment.

*E. coli* is the most preferred choice as expression host, because it is cultured and genetically manipulated easily, grown fast, and fermented simply [9]. Different *E. coli* strains are used for producing soluble and functional proteins under control of the P_T7lac_ promoter in the pET expression plasmids. Among them, the BL21(DE3) strain is frequently utilized. The Rosetta (DE3) cells harbor the plasmids for coexpressing several rare tRNAs to increase heterologous protein expressivity, whereas the BL21(DE3)pLys cells carry the plasmids for coproducing the T4 lysozyme as the T7 RNA polymerase inhibitor to block background expression of the protein of interest [10]. The Origami(DE3) strain is often used for producing disulfide-rich protein in the cytoplasm, since genes encoding thioredoxin reductase, glutathione reductase, and alkyl hydroperoxide reductase subunit C in cytoplasmic reductive pathways are knocked out, facilitating the disulfide bonds formation of the recombinant protein [11]. Previously, we combined the reported five mutations for improving protein solubility [3, 4], and S219V to construct the TEVp variant, called TEVp^5M^ [12]. Specific proteolysis of our designed fusion protein by the TEVp construct affords the tag-free diaminopropionate ammonia-lyase (DAL) with significantly increase in the catalytic activity, as compared to the tagged DAL [13]. In BL21(DE3) and Rossetta(DE3) strains, we overexpressed four TEVp variants and identified that desirable soluble production, the cleavage activity, and protein stability are not combined [12].

Biopharmaceutical production of disulfide-rich protein in *E. coli* cytoplasm usually forms inactive inclusion bodies (IBs), which are refolded to form soluble active protein. Several fusion tags have been documented to enhance refolding efficiency [14]. The yields of the refolded proteins are obviously increased by use of on-column refolding method through the fused affinity tag for immobilizing the specific resin [15]. The C-terminally attached cellulose-binding module (CBM) rendered renaturation of the disulfide-rich single-chain antibody partner on the cellulose with the increased soluble amounts from IBs [16]. Regenerated amorphous cellulose (RAC) displays higher binding capacity of the CBM tag than the cellulose [17]. To assist the disulfide bonds formation during refolding of disulfide-rich proteins, the buffer is prepared at the specified ratio of the reducible/oxidizable chemical pairs, including glutathione (GSH) and disulfide-bonded glutathione (GSSG), cysteine and disulfide-bonded cystine to induce the oxidative redox state [18]. However, the reagent with the changed redox potential can result in cysteine oxidation. So, mutations of cysteine residues, selection and optimization of TEVp variants with the combined desirable yields, cleavage activity, and oxidative stability are analyzed by trial and error, like the identification of the other TEVp mutants with higher cleavage activity and improved thermostability [19-21].

In this work, we introduced C19S, C110S and C130S in the TEVp^5M^ and combined mutations to construct seven variants, and overexpressed the constructs in four *E. coli* strains, and the analyzed protein solubility and cleavage activity. The current results showed that C110S and C130S improved oxidative stability of the TEVp^5M^, whereas C19S resulted in protein mainly produced as IBs. In the buffer containing the chemical pairs for forming the oxidative redox state, purified TEVp^5M^C110S/C130S exhibited the highest cleavage activity among the selected variants. It cleaved the fusion protein for the CBM tag immobilized on the RAC to release the non-tagged active bovine enterokinase catalytic domain (bEK) and maize peroxidase (mPex), after RAC matrix-assisted refolding.

## Materials and Methods

### Bacterial Strains, Plasmids and Reagents

*E. coli* strains DH5α, BL21(DE3), BL21(DE3)pLys, Rossetta(DE3) and Origami(DE3), and the plasmids pET-22b and pET-28b are products of Novagen (USA). The pET41a-EK vector was gifted from Professor Zhao Zhongbao [22]. Several plasmids were constructed in our laboratory [13, 14, 23], including pET28-TEVp^5M^ for expressing the double His6-tagged TEVp^5M^, and pET28-EmGFP containing the insertion encoding the fused emerald GFP (EmGFP), pGST-eDAL or pGST-sDAL plasmid encoding the His6-tagged glutathione S-transferase (GST) fused to DAL from *E. coli* (eDAL) or *Salmonella* typhimurium (sDAL), pCBM-tevS to express the CBM tag fused to the linker GGTGGS around the tevS. All proteins are produced under control of the P_T7lac_ promoter. The MutanBEST Kit for site-directed mutagenesis, and reagents for plasmid construction and protein overexpression were supplied by Takara (China). Nickel-nitrilotriacetic acid (Ni-NTA) agarose was provided by Qiagen (USA). Ultra-15 centrifugal ﬁlter tube and Ultracel-10 membrane were made by Amicon (USA). Mouse anti-His6 monoclonal antibody and horseradish peroxidase (HRP)-conjugated anti-mouse IgG antibody were purchased from GenScript (China). Reagents for analyzing the DAL activity such as DL-α,β-diaminopropionate (DL-DAP), pyridoxal 5’-phosphate (PLP), 2,4-dinitrophneylhydrazine (DNP) and *o*-phenylenediamine (OPA) were bought from Sigma (USA).

### Plasmids Construction

The primer pairs C19S1 and C19S2, C110S1 and C110S2, and C130S1 and C130S2 were designed for site-directed mutation of the TEVp^5M^ (Table S1) by PCR using the plasmid pET28-TEVp^5M^ as the template. After it was sequenced, the fragment encoding the TEVp construct was excised with NcoI and XhoI and subcloned into NcoI-SalI site of the pET28-EmGFP plasmid to express the TEVp variant fused to the EmGFP. Considering the Origami(DE3) strain’s resistance to kanamycin, the XbaI -XhoI excised fragment was subcloned into the pET-22b plasmid with the same restriction enzyme treatment to confer the transformants with ampicillin resistance.

The amino acid sequence of the HRP was compared with that of the mPerx (Fig. S1). The fragment encoding the leaderless mPex Q45-S350 was amplified by RT-PCR using the total RNAs extracted from maize leaves as the template, and primers mPex1 and mPex2. The PCR amplicon was incubated with BamHI and XhoI, and inserted into the pCBM-tevS vector with the same treatment to yield the pCBM-mPerx vector for expressing the CBM-tevS-mPerx. The plasmid to overexpress the CBM tagged bEK was constructed by excising the sequence encoding the bEK from the plasmid pET41a-EK with BamHI and XhoI, and inserting into the pCBM-tevS plasmid treated in the same manner. All constructed plasmids were sequenced to determine correction.

### Production of the TEVp Variants in Different *E. coli* Strains

The recombinant cells were cultured overnight at 37°C in 5 ml of lysogeny broth (LB), diluted to 100-fold and grown at 37°C in 10 ml liquid culture of a 50-ml shake flask at 220 rpm. The target protein was induced at 28°C for 12 h by use of 0.5 mM isopropylthio-β-D-galactoside (IPTG) at OD_600_ of about 0.5, as measured on a U-2900 spectrometer (Hitachi, Japan). After centrifugation (4,000 *g*, 10 min, 25°C), the collected cells were washed and re-suspended with buffer A (20 mM Tris-HCl, pH 8.0, 100 mM NaCl). With sonication at 4°C followed by centrifugation (12,000 *g*, 15 min, 4°C), soluble protein samples were prepared. The precipitates were washed twice with buffer A, re-suspended with buffer A containing 8 M urea at room temperature for 2 h to solubilize the aggregated proteins, and centrifuged (12,000 *g*, 15 min, 25°C) to remove cell debris. Protein concentration was determined by the Bradford method, based on the relationship between absorption at 595 nm and the prepared bovine serum albumin solutions. Protein samples were analyzed by sodium dodecyl sulfate polyacrylamide gel electrophoresis (SDS-PAGE). Western blotting was performed by transforming the separated proteins on the SDS-PAGE gel to polyvinylidene ﬂuoride (PVDF) membrane, blotting with anti-His6 monoclonal antibodies, and treating with the HRP-conjugated goat anti-mouse IgG. The target protein was observed on the PVDF membrane after 4-chloro-1-naphthol and 0.08% H_2_O_2_ were added.

### Solubility Analysis of the TEVp Variants

The C-terminally fused EmGFP reporter was used for quantitative analysis of the soluble TEVp amounts, based on the measurement of the soluble fusion proteins for the EmGFP on a F-4500 fluorescence spectrometer (Japan). The excitation and emission maximums of the EmGFP are 488 and 515 nm [13].

### Coupled Assay of the Cleavage Activity

The His6-tagged GST-tevS-eDAL as the TEVp substrate was purified by Ni-NTA matrix [12]. Purified protein substrate and soluble extracts containing the recombinant TEVp construct were mixed at mass ratio of 30:1, whereas purified proteins including GST-tevS-eDAL and TEVp construct were added at mass ratio of 50:1. The cleaving reaction occurred at 30°C for 1 h to ensure the protein substrate was partly degraded by the TEVp at the specified mass ratio. The TEVp cleavage activity was estimated by the coupled assay. As a PLP-dependent enzyme, DAL transforms DL-DAP into pyruvate and ammonia. For the DAL activity assay, 1 ml mixture containing 50 μM PLP, 10 mM DL-DAP, and the eDAL were incubated at 37°C for 5 min. To stop the reaction, 1 ml of 2M HCl and 0.03% DNP was added. The solution was placed at 4°C for 5 min, and 2 ml of 2 M NaOH was added. After centrifugation (12,000 *g*, 10 min, 25°C), absorbance at 520 nm representing pyruvate amounts in soluble fraction was measured.

### Purification of the TEVp Variants

The expression plasmids were transformed into the BL21 (DE3) cells and proteins were induced at 28°C for 12 h in 500 ml LB culture. After centrifugation, recombinant cells were washed with buffer B (50 mM sodium phosphate, pH 8.0, 300 mM NaCl, and 10 mM imidazole), disrupted and centrifuged. The clear lysate was loaded on a column containing 4 ml Ni-NTA resin pre-equilibrated with 40 ml buffer B, and washed twice with 40 ml buffer B (pH 8.0) containing 40 mM imidazole. The constructed TEVp protein was eluted with 40 ml buffer B (pH 8.0) containing 250 mM imidazole. Purified protein was concentrated in the Ultra-15 centrifugal filter, and simultaneously exchanged with buffer A using the Ultracel-10 membrane, and stored at -80°C.

### Refolding of the Fusion Proteins and Release of the Target Enzymes via Tag Removal

The IBs from the BL21(DE3) cells carrying the plasmids encoding the CBM-tagged bEK or mPex were collected, washed and re-suspended with buffer C [30 mM Tris/HCl, 150 mM NaCl, 10% (v/v) glycerol, 0.5% (v/v) Triton X-100, pH 7.5]. Then, IBs were re-suspended with buffer C in the absence of Triton X-100. The tagged bEK in the prepared IBs was solubilized with buffer D (30 mM Tris-HCl, 200 mM NaCl, 8 M urea, 5 mM DTT, and 10 mM EDTA-Na_2_). To solubilize sufficient amounts of IBs, the mis-matched disulfide bonds in the proteins were abolished in the presence of DTT, and the mixture was incubated at room temperature for 2 h, and centrifuged (18,000 *g*, 30 min, 25°C) to remove the pellet. The mPex construct in the IBs was solubilized with buffer E (40 mM Tris-HCl, pH 9.0, 4.5 M urea, 5 mM DTT) to a protein concentration of 0.3 mg/ml, according to the published report with slight modification [24]. The mixture was centrifuged (18,000 *g*, 30 min, 25°C) and the solubilized protein was collected.

To increase the amounts of the CBM tag above that of the cellulose, RAC was prepared, as in a previously described procedure [17]. For the bEK refolding [25], the protein was diluted with buffer F (100 mM Tris–HCl, 6 M urea, 10 mM cystine, pH 8.0), and RAC was added. The mixture was diluted slowly with buffer G [80 mM Tris–HCl, 0.7 M urea, 15% (v/v) glycerol, 0.5 mM cysteine, 5 mM cysteine, 2 mM CaCl_2_]. For the mPex refolding [24], the denatured proteins were diluted with buffer H [40 mM Tris-HCl, pH 8.5, 0.5 M urea, 5% glycerol (V/V), 2 μM hemin, 2 mM CaCl_2_, 0.5 mM GSH and 5 mM GSSG]. After the refolding process was finished, the mixture was centrifuged (3,000 g, 10 min, 25°C), the resin was washed three times with buffer G for the bound bEK, or four times with buffer H in absence of hemin for binding to the constructed mPex. Purified TEVp variant, M6 protein (Tab.1), was incubated with the refolded protein bound to RAC with mass ratio of 1: 10 at 10°C for 24 h. Then, Ni-NTA resin was added and incubated for 2 h at room temperature. Followed by centrifugation (3,000 *g*, 10 min, 25°C), the supernatant was collected. Prepared protein samples were subjected to SDS-PAGE analysis.

### Activity Assay of the Refolded Tagless bEK and mPex

The non-tagged bEK cleaving the GST-tagged sDAL with incorporation of the D4K as the bEK recognition sequence was analyzed by the coupled assay of sDAL activity, because bEK can cleave eDAL at the undesired site to inactivate the eDAL [14]. The activity assay of sDAL was the same as that of eDAL. Like HRP, mPex catalyzes H_2_O_2_ degradation. Using OPA as a hydrogen donor, the reaction mixture turns yellow upon oxidation [26]. The freshly prepared tag-free mPex was incubated in buffer I (20 mM Tris-HCl, pH 7.5, 50 μg/ml OPA, 10 mM or 30 mM H_2_O_2_) at 37°C for 30 min, and absorption at 411 nm was measured.

### Statistical Analysis

Data were indicated as means ± standard deviations (SD), evaluated using a one-tailed *t*-test, and analyzed using SPSS ver. 22 (SPSS Inc., USA).

## Results 

### Design of the Fusion Proteins

**Fig. 1:**
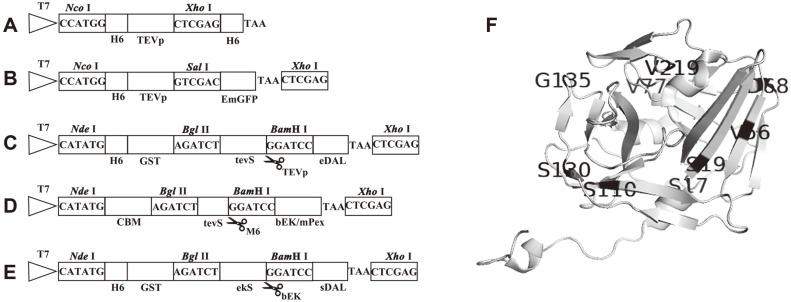
Design of fusion proteins. All target genes were cloned in frame downstream of the P_T7lac_ promoter in the pET expression plasmids. The restriction enzymes are indicated above the genes, and the fusion constructs were schematically represented below the genes (not to scale). (**A**) The double His6-tagged TEVp^5M^ contains T17S, L56V, N68D, I77V, S135G, and S219V. Each and combined C19S, C110S and C130S were introduced into the TEVp^5M^, respectively. (**B**) The constructed TEVp was fused to the EmGFP for detecting production level in soluble extracts. (**C**) The His6-GST tagged eDAL for the TEVp cleavage. Between the His6-GST and eDAL, the tevS representing as the ENLYFQ↓G encoded by the sequence including BglII and BamHI cut sites was incorporated. (**D**) The sequence encoding the CBM-tagged bEK or mPex. The tevS was introduced between two protein partners. After refolding, the fusion protein was cleaved by the M6 variant for releasing the target protein. (**E**) The His6-GST tagged sDAL as the bEK substrate. As shown in Fig. 1C, the placed tevS was substituted with the ekS. (**F**) The mutational amino acids in the TEVp are based on the crystal structure (Protein Data Bank code 1LVM). The figure was created with the program Pymol (https:pymol.org).

In this work, we constructed different expression plasmids. The double His6-tagged TEVp constructs were used for SDS–PAGE-based analysis of protein solubility and rapid purification (Fig. 1A). The constructed TEVp mutants were named, as listed in Table 1. Three constructs include M1-M3 with one cysteine mutation in the TEVp^5M^. The M4–M6 constructs contained two mutated residues, and M7 included three mutations. For quantitative analysis of protein solubility, we fused the TEVp to the EmGFP, as presented in Fig. 1B, like our earlier work [12]. The fusion protein as the TEVp substrate was constructed previously, including the His6-tagged GST, the tevS and eDAL (Fig. 1C). Two disulfide-rich enzymes independently fused to the CBM tag and the tevS were overexpressed in *E. coli* BL21(DE3), refolded from IBs and released from the immobilized on RAC with purified TEVp M6 construct constructed in this work (Fig. 1D). For assaying the bEK cleavage activity, the bEK recognition sequence D4K↓G (ekS) was incorporated between His6-GST and sDAL (Fig. 1E). Based on the TEVp crystal structure, C110S and C130S are relatively far from the mutations for augmenting soluble production and S219V for inhibiting auto-cleavage, different from C19 (Fig. 1F).

### Production of the TEVp Variants in Different *E. coli* Strains

**Fig. 2:**
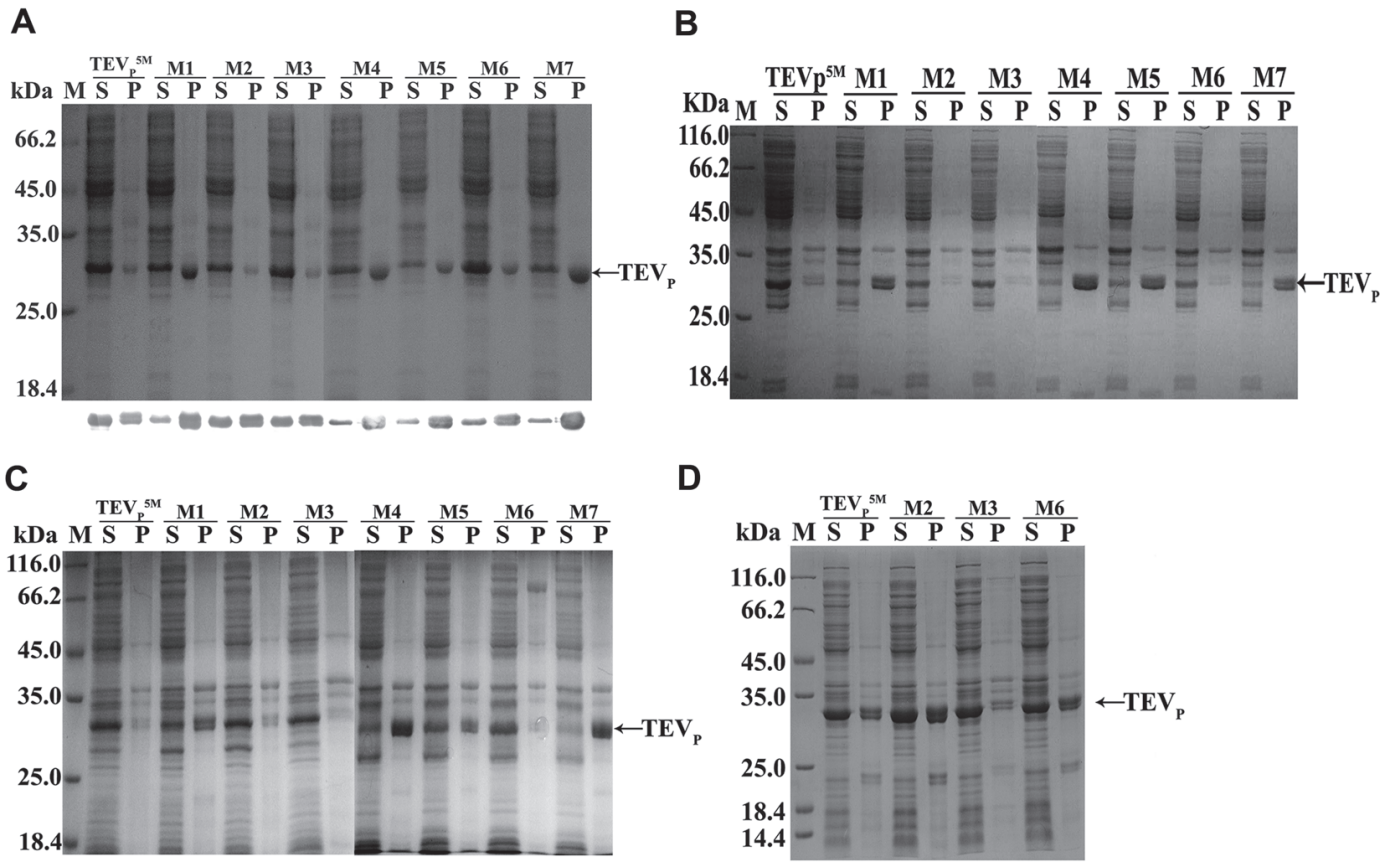
Production of the constructed TEVp variants in the BL21(DE3) (**A**), BL21(DE3)pLysS (**B**), Rosetta (DE3) (**C**) and Origami(DE3) (**D**) strains. About 10 μg of soluble proteins, and 4 μg of proteins in pellets were separated by SDS-PAGE. In the Fig. 2A, the specific bands detected by using anti-His6 monoclonal antibodies for Western blot analysis were shown on bottom of the gel. M: protein marker. S: soluble. P: pellet. Variant names are denoted on the top of the gel. Arrows indicated the position of the TEVp constructs.

Four *E. coli* expression strains were selected to explore the effects of the introduced mutations on the yields, protein solubility, activity, and the oxidative stability. They are widely used for producing recombinant proteins with desirable production and functionality. As detected by SDS-PAGE (Fig. 2A), the M1, M4, M5 and M7 containing C19S were less soluble than the TEVp^5M^ in *E. coli* BL21 (DE3) cells. The soluble and insoluble fractions contained the TEVp construct, as detected by Western blotting (Fig. 2A). Soluble production of the TEVp^5M^ was decreased by C19S, which was not recovered by further C110S and/or C130S. In the BL21(DE3) pLysS, four variants containing C19S also showed less solubility than the TEVp^5M^ (Fig. 2B). In the Rossetta(DE3), soluble production of the constructs was not obviously increased (Fig. 2C), although several rare codons are distributed in the TEVp coding sequence, for example, the arginine codons AGA and AGG [27]. Because C19S decreased protein solubility, we expressed the other three TEVp variants in the Origami(DE3). The variant protein solubility was augmented, compared with that of the TEVp^5M^ (Fig. 2D). The results suggested that C110S and C130S conferred the TEVp^5M^ with the improved oxidative stability.

### Determination of Protein Solubility

**Fig. 3:**
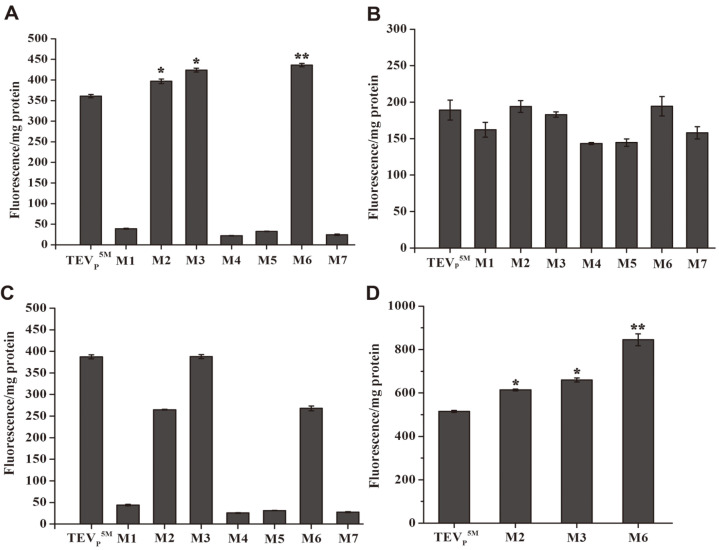
Soluble expression levels of the TEVp constructs in the BL21(DE3) (**A**), BL21(DE3)pLysS (**B**), Rosetta (DE3) (**C**) and Origami(DE3) (**D**) strains determined by using the EmGFP reporter. At least five samples were measured under the same induction conditions, and the closest values were counted and indicative of means and standard deviation. The asterisk indicated significant differences higher than the TEVp^5M^ as the control; * *p* < 0.01.

The fused GFP is often applied for determining the protein solubility of the target proteins [28]. Using the C-terminally fused EmGFP reporter, we analyzed the constructed TEVp solubility quantitatively in supernatants. Introduction of C110S and/or C130S increased the TEVp^5M^ solubility in the BL21(DE3) cells, in contrast to that of C19S (Fig. 3A). The results were similar to the observation of the TEVp constructs on the SDS-PAGE gel (Fig. 2A). The soluble amounts of certain constructs were comparable to those of the TEVp^5M^ in the BL21(DE3)pLys (Fig. 3B), suggesting that inhibition of basal expression assisted the variant protein folding. Supply of rare tRNAs hardly increased soluble production of all variant amounts, except for the TEVp^5M^ (Fig. 3C), suggestive of each mutation potentially impairing protein folding. In the Origami(DE3), soluble production of the variants containing C110S and/or C130S was higher than the TEVp^5M^, probably due to the improved oxidative stability (Fig. 3D). SDS-PAGE analysis also showed that soluble production of the GFP-tagged TEVp^5M^ and the constructs containing C110S and/or C130S were changed in different *E. coli* strains (Figs. S2, A-D). All fusion constructs were observed to have retained stability in the cytoplasm.

### Cleavage Activity of the TEVp Variants in Clear Lysates

**Fig. 5:**
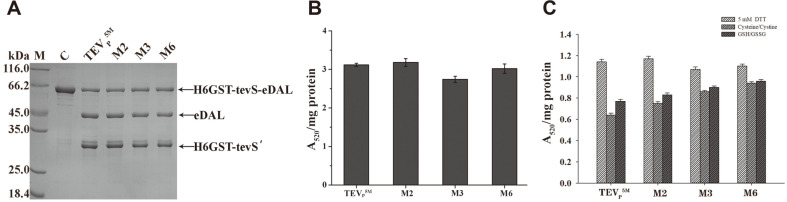
In vitro analysis of the cleavage activity of purified TEVp variants’ proteins. (**A**) SDS-PAGE analysis of the fusion protein after TEVp cleavage. Names of the TEVp variants used for the cleavage are denoted on the top the gel. Arrows indicated fusion protein and the cleaved products. The His6-tagged GST fused to partial tevS as the cleaved product was denoted as H6GST-tevS’. (**B**) The cleavage activity of the purified TEVp variants’ proteins by the coupled assay. (**C**) The cleavage activity of purified TEVp constructs in the presence of 5 mM DTT, or 5 mM cystine plus 0.5 mM cysteine, or 5 mM GSSG plus 0.5 mM GSH. The asterisk indicated significant differences higher than the TEVp^5M^ as the control; * *p* < 0.01.

Since the fused GFP reporter possibly affects the oxidative stability of the TEVp variants, we selected the His6-tagged TEVp variants for testing the cleavage activity. Each of the TEVp^5M^, M2, M3 and M6 variants in supernatants from four *E. coli* expression strains cleaved purified GST-tevS-eDAL partially, more efficiently than the other ones (Figs. S3A-S3D). Among the mutants, the M3 in the BL21(DE3), TEVp^5M^ in BL21(DE3)pLysS and Rossetta(DE3), and the M6 in Origami(DE3) showed the highest activity (Figs. 5A-5D). On the other hand, the M6 was most soluble in the BL21(DE3), BL21(DE3)pLysS and Origami(DE3), and the TEVp^5M^ and the M3 showed higher soluble production than the other variants in Rossetta(DE3) cells (Figs. 3A-3D). The results indicated that, yield, protein solubility, the activity and oxidative stability are divergent features, and all parameters cannot be concomitantly enhanced in the recombinant TEVp. The correlation among the characters analyzed in the study provides a consistent view of mutational effects on the TEVp constructs.

### Effect of the Oxidized Redox State on the Activity of Purified TEVp Constructs

The purified TEVp variants’ proteins cleaved the partial fusion protein, as detected by SDS-PAGE (Fig. 5A). The specific activity of the TEVp constructs was comparable to the TEVp^5M^, but the M3 activity was slightly decreased (Fig. 5B). With addition of 2 mM DTT, three constructs showed cleavage activity comparable with the TEVp^5M^. In contrast, addition of chemical pairs including 5 mM cystine and 0.5 mM cysteine, or 5 mM GSSG and 0.5 mM GSH inhibited the TEVp^5M^ activity, but the M6 mutant displayed least sensitivity to the oxidative redox in buffer (Fig. 5C), most likely attributed to C110S and C130S.

### Removal of Fusion Tags in Two Refolded Proteins Containing Multiple Disulfide Bonds

**Fig. 6:**
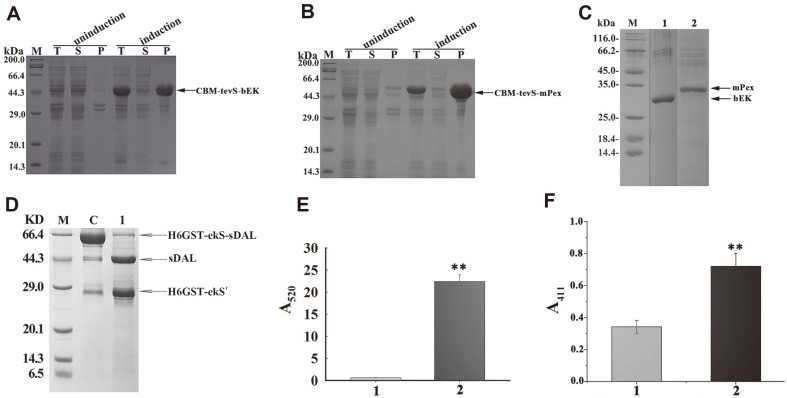
Overexpression, refolding, tag removal, and activity assay of the disulfide-bonded bEK and mPex. A: SDS-PAGE analysis of the CBM-tagged bEK produced in *E. coli* BL21(DE3) strain. T: total protein. S: soluble fraction. P: insoluble fraction. B: SDS-PAGE analysis of the CBM-tagged mPex. C: the refolded tag-free bEK and mPex released from RAC resin with the purified M6 incubation. Lane 1: the refolded tag-free bEK. Lane 2: the refolded tag-free mPex. Arrows indicate the two purified enzymes. D: Cleavage of purified His6-tagged GST-ekS-sDAL with the refolded bEK. Lane C: the fusion protein incubated with the heat-inactive bEK. Lane 2: the fusion protein incubated with the refolded tag-free bEK. Arrows indicate fusion protein substrate and cleaved products. The His6-tagged GST fused to partial ekS as the cleaved product was denoted as H6GST-ekS’. E: The coupled assay of the refolded tag-free bEK activity. Absorption from the mixture in the absence and presence of the refolded tag-free bEK is indicated as light and dark grey columns. F: Activity of the mPex catalyzing 10 mM H_2_O_2_ degradation. Absorption from the mixture in the absence and presence of the refolded tag-free mPex is indicated as light grey and black columns. The asterisk indicates significant differences higher than the inactivated enzyme as the control; *p < 0.01.

Two proteins as the fusion partner were chosen for refolding while M6 with the improved oxidative stability was used for removing the fused CBM tag. The bEK and mPex as fusion carriers were produced in *E. coli* BL21(DE3) mainly as inclusion bodies (Figs. 6A and 6B). The fusion protein was refolded on RAC via the CBM tag binding, and the target protein was released from the resin by using purified M6 protein digestion (Fig. 6C). The yields were about 0.7 mg for the renatured bEK and 3.1 mg for the refolded mPerx from 100 ml LB culture. As comparison, about 3.8 ± 1.1 mg bEK proteins after refolding and purification with benzamidine affinity chromatography are obtained from 1 L of culture [25], and 1.8 mg of HRP proteins are refolded from 100 ml LB media [24]. The refolded bEK cleaved the GST-tagged sDAL into two parts, as shown on the SDS-PAGE gel (Fig. 6D). After cleavage, the released sDAL displayed higher catalytic activity, as detected by DNP reacting with pyruvate for pigmentation (Fig. S4A), and was measured by colorimetric analysis (Fig. 6E). With addition of the refolded mPex, 10 and 30 mM H_2_O_2_ were partially transformed into yellow compounds in the presence of OPA (Fig. S4B), and colorimetric assay showed the refolded mPex was catalytically active toward 10 mM H_2_O_2_ (Fig. 6F), in contrast to the heat-inactivated mPex. The other fusion tag has been previously applied for increasing renaturation efficiency, for example, the small molecular chaperone [29]. So, the M6 variant is an ideal alternate for tag removal in the refolding mixture under oxidative environment.

## Discussion

In this study, we introduced and combined C19S, C110S and C130S in the TEVp^5M^, and identified that desirable protein productivity, solubility, cleavage activity, and oxidative stability were not concurrently favored in the recombinant *E. coli* cells. The intracellular process of the fusion protein in *E. coli* is completed by coexpressing the TEVp [30]. Owing to higher production and activity of the M6 variant in the Origami(DE3), we believe that the coexpressed M6 will hopefully increase cleavage efficiency in the oxidative cytoplasm generated in other strains, such as Rosetta-gami(DE3) and Shuffle T7 [11], or by coproducing each of different thiol oxidase constructs [31]. It is noted that, beside the cysteine, methionine is also oxidized reversibly to methionine sulfoxide under strong oxidative stress [32]. TEVp contains eight methionine residues. Further study will focus on the optimizations of methionine mutation in the M6 variant.

C19, C110 and C130 are located in the first, eighth, and tenth β-strands in the TEVp protein structure respectively [7,8]. The C19 buried in the TEVp is deduced to be reluctant to form the uncorrected disulfide bridge with another TEVp molecule. The C151S transforms the TEVp into serine protease, but soluble production of the TEVp variant in *E. coli* requires other mutations [33]. Introduction of C110S and C130S to the TEVp variant as the serine protease is expected to further enhance the oxidative stability. Both mutations will be also combined with the other mutated residues in the TEVp contributing to enhanced thermostablility and catalytic efficiency [19-21]. TEVp is not versatile for tag removal and exhibits poor ability to cleave the fusion construct bound to *E. coli* membrane [34]. The current work provides a possible approach to create new TEVp variants with increased hydrophobicity to overcome this limitation.

The CBM tag absorbed with the RAC resin probably minimizes the contact between target protein molecules, thus preventing their aggregation. This tag maintains the binding stability in the refolding buffer containing 6 M urea [16]. In this work, the refolded bEK and mPerx with the CBM tag bound to the RAC were used as immobilizates for improving use efficiency and easy extraction from the reaction mixture. Incubation of the purified M6 allowed the target proteins to be released from the affinity matrix. So far, different TEVp variants have been created for improving protein folding, solubility, thermostability, and catalytic activity [2, 12, 19-21, 35]. Our work expands the TEVp toolkit, and the M6 variant is used for generating refolded tag-free bEK and mPex with biotechnological and medical values [24, 25].

In summary, the multiple mutations conferred the TEVp with enhanced solubility, poor self-inactivation, and improved oxidative stability. Cleavage of the immobilized fusion proteins with purified M6 protein in the buffer under oxidative redox state mediated release of the refolded disulfide-rich proteins from the prepared RAC as an affinity adsorbent.

## Supplemental Materials

Supplementary data for this paper are available on-line only at http://jmb.or.kr.

## Figures and Tables

**Fig. 4:**
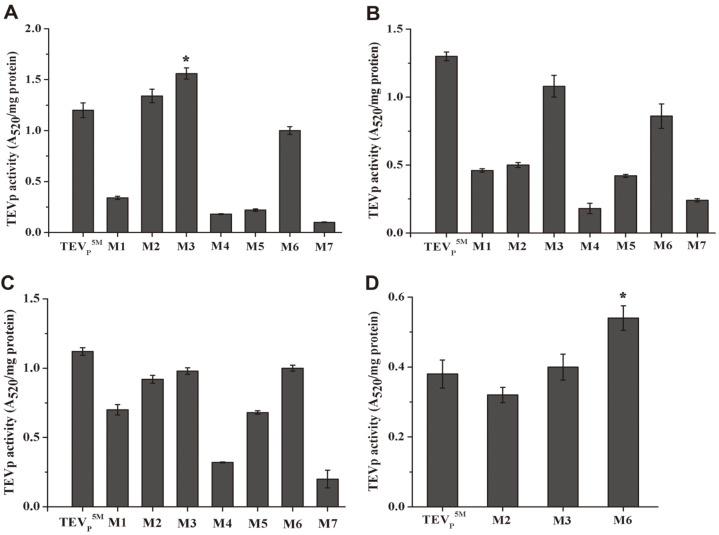
The coupled assay of the cleavage activity of the TEVp^5M^ or the variant produced in the BL21(DE3) cytoplasm (**A**), BL21(DE3)pLysS cells (**B**), Rosetta(DE3) host (**C**) and Origami(DE3) strain (**D**). Data are means and standard deviation of three technical replicates. The heat-inactivated TEVp construct was used as control, and the absorption was subtracted. The asterisk indicated significant differences higher than the TEVp^5M^ as the control; * *p* < 0.01.

**Table 1:** The variants constructed in this study.

M1	M2	M3	M4	M5	M6	M7
TEVp^5M^	TEVp^5M^	TEVp^5M^	TEVp^5M^	TEVp^5M^	TEVp^5M^	TEVp^5M^
+C19S	+C110S	+C130S	+C19S/C110S	+C19S/C130S	+C110S/C130S	+C19S/C110S/C130S
