# Supplementary material for: Construction, Investigation and Application of TEV Protease Variants with Improved Oxidative Stability
Source: J Microbiol Biotechnol. 2021 Sep 10;31(12):1732–40. doi: 10.4014/jmb.2106.06075 (PMC9705859; doi:10.4014/jmb.2106.06075)
Supplement: Supplementary file 1 [file jmb-31-12-1732-supple.pdf]

J. Microbiol. Biotechnol.
https://doi.org/10.4014/jmb.2106.06075

Supplementary Materials

Table S1. The primers used in this study

Primer	Sequence(5'-3')
C19S1	F: GATATCGAGCAGCATTAGTCATTTGACGAATG
C19S2	R: CATTCGTCAAATGACTAATGCTGCTCGATATC
C110S1	F: GTGAAGAGCGCATTAGTCTTGTGACAACCAAC
C110S2	R: GTTGGTTGTCACAAGACTAATGCGCTCTTCAC
C130S1	F:GTGTCAGACACTAGTAGCACATTCCCTTCAG
C130S2	R:CTGAAGGGAATGTGCTACTAGTGTCTGACAC
mPex1	F: CTCGGATCCCAACTCATGGTCGGATACTACAAG
mPex2	R: CAGTCTCGAGTTAGGACCTGCTGTTGACCTTGC

F：forward primer. R: reverse primer. The restrictive enzyme cut sites are indicated by the underlines. The mutated bases in the forward primers are marked in red.

```
mHRP            --------------------------------------------QLTPTFYDNSCPNVSN
mPex            MTTSKLAVLTLFALLGSVSCQSGGYYFYPTPQQPSPTPSPPSSPQLMVGYYKDKCAAYVD
                                                            **   :*.:.*.   :

mHRP            IVRDTIVNELRSDPRIAASILRLHFHDCFVNGCDASILLD---NTTSFRTEKDAFGNANS
mPex            VEAIVKKHVKATDAGMQAGLVRLLFHDCFVRGCDGSVLLDTFSNDTSLTPEKFGVPNFPS
                :   .  :   :*. : *.::** ******.***.*:***   * **: .** .. *  *

mHRP            ARGFPVIDRMKAAVESACPRTVSCADLLTIAAQQS--VTLAGGPSWRVPLGRRDSLQAFL
mPex            LRGFEVIDAAKAEIEAACPGTVSCADIVAFAARDASYFLSGGGISFAMPAGRYDGNVSLA
                 *** ***  ** :*:*** ******::::**:::  .  .** *: :* ** *.  ::

mHRP            DLANANLPAPFFTLPQLKDSFRNVGLNRSSDLVALSGGHTFGKNQCRFIMDRLYNFSNTG
mPex            SETLPNLPSPFTGFDQLVKVFADKGLD-AFDMITLSGAHSIGRSHCSSFTRDRLPPSNTT
                . : .***:**  : ** . * : **: : *:::***.*::*:.:*  :       ***

mHRP            LPDPTLNTTYLQTLRGLCPLNGNLSALVDFDLRTPTIFDNKYYVNLEEQKGLIQSDQELF
mPex            DIDP----AFAATLQASCASPNGTDNTVMQDFKTPDVLDNQYYKNVLAHKVLFTSDAALT
                  **    ::  **:. *.  .. .  *  *::** ::**:** *:  :* *: **   *

mHRP            SSPNATDTIPLVRSFANSTQTFFN-AFVEAMDRMGNITPLTGTQGQIRLNCRVVNSNS
mPex            TNFTSNN---LVRAYADFVPYLWQQKFAKAMVKMAGVEIKTAANGEIRKTCRKVNSRS
                 . .:.:   ***::*: .  :::  *.:** :*..:   *.::*:** .** ***.*
```

Fig. S1. Alignment of the deduced amino acid sequences of mature HRP (mHRP) with mPerx. Identical amino acid residues are represented by asterisks. The alignment was performed using CLUSTALW (https://www.genome.jp/tools-bin/clustalw).

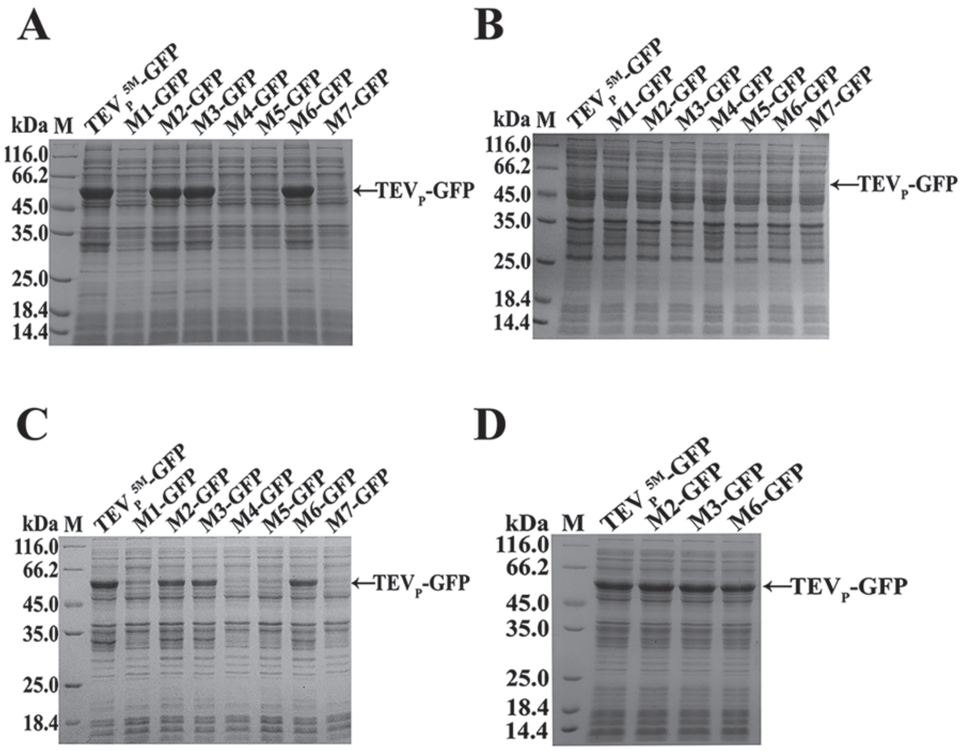

Figure S2 SDS-PAGE analysis of the C-terminally EmGFP tagged TEVp^{5M} and its variants. About 10 μg soluble proteins expressed in BL21(DE3) (a), BL21(DE3)pLysS (b), Rosetta (DE3) (c) and Origami (DE3) (d) were separated by 12% SDS-PAGE.

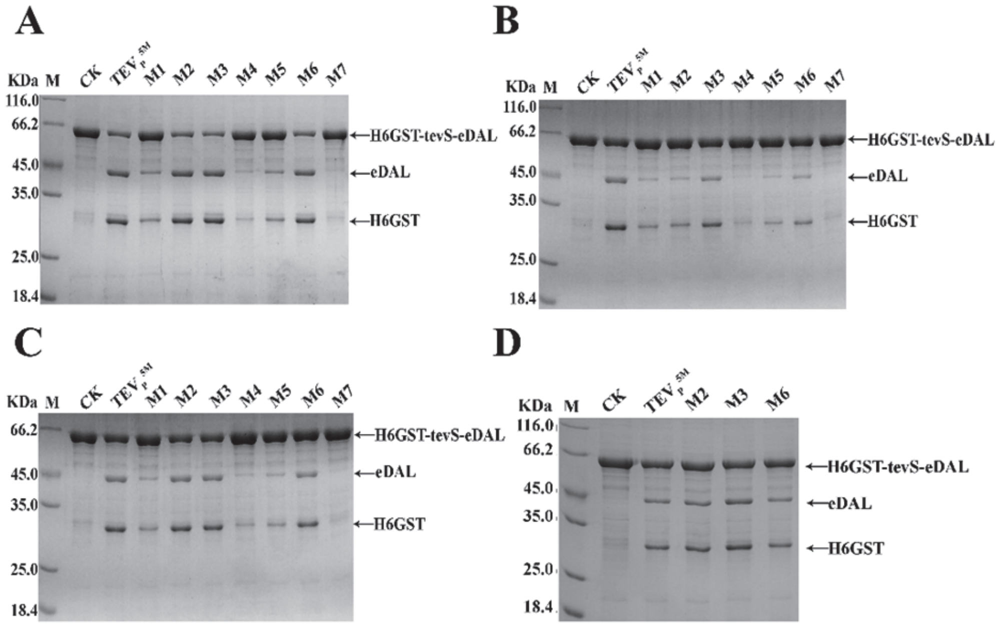

Figure S3 SDS-PAGE analysis of the purified fusion protein incubating with the TEVp construct in crude extract. CK: the fusion protein incubating with the soluble extract from the correspondent cells carrying the pET-28b or pET-22b plasmids. The fusion protein substrate and cleaved products were indicated by arrows.

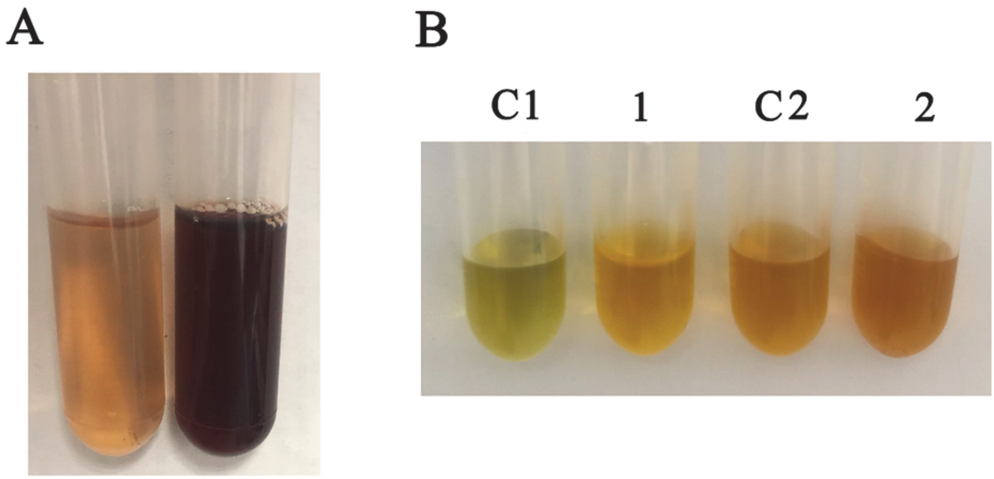

Figure S4 The activity assay of the bEK and mPex. A: the refolded tag-free bEK cleaved the fusion protein for the sDAL, rendering the released sDAL with the obviously increased activity for transformation of DL-DAP and coloration of pyruvate as the catalytic product with DNP. B: The activity assay of the refolded tag-free mPex. C1 and C2 represented the reaction mixture containing 10 mM or 30 mM H_2O_2 with addition of the heat-inactive mPerx (100 °C for 10 min). 1 and 3 represented the mixture with addition of the refolded mPex and OPA. Activity assays of the bEK and mPex were described in Material and Methods section.
